# Self-direction in Productive Aging: A Qualitative Study

**DOI:** 10.1093/geroni/igaf122.2762

**Published:** 2025-12-31

**Authors:** Marilyn Cole, Karen Macdonald

**Affiliations:** Quinnipiac University, Stratford, Connecticut, United States; Sacred Heart University, Fairfield, Connecticut, United States

## Abstract

In this qualitative study by two occupational therapy researchers, our goal was to explore the perspectives of healthy productive agers, actively engaged in their communities. Researchers interviewed six retired older adults, ages 66-86. Using qualitative analysis, they identified three themes: hope for the future, planned performance, and purposeful social participation. Hope for the future includes a positive anticipation of future events. In order to set positive future goals, participants spoke of cognitive, emotional, and spiritual attributes. They demonstrated self-efficacy in managing their own health and some serious chronic conditions in order to maintain their meaningful personal and social activities. Planned performance required high cognitive skills in the realistic use of time and resources to accomplish needed self-care, home maintenance, and for some, caregiving responsibilities. Purposeful social participation was a dominant theme for participants who voluntarily took on many responsible social and community roles in retirement. It surprised researchers that each person valued their memberships in social groups so highly, whether families, neighborhoods, common interest groups, or charitable and religious organizations. This study highlights the importance of enabling self-direction for older adults. Given the choice and resources, each participant found ways to live productively. This guides professional service providers and policy makers to recognize the strong self-directed sense of purpose and mastery of self-management needed by older adults who seek to lead meaningful and productive lives, and to contribute to their communities and society.

